# Consult, Connect, Collaborate: Cross-Sector Approach to Recruitment and Retention in Psychiatry

**DOI:** 10.1192/bjo.2024.308

**Published:** 2024-08-01

**Authors:** Nina MacKenzie, Alastair Cook

**Affiliations:** 1 Scottish Government, Edinburgh, United Kingdom; 2 NHS Lanarkshire, Motherwell, United Kingdom

## Abstract

**Aims:**

Mental ill-health is one of Scotland's most pressing challenges, with an expectation of increased workload for health services. There are substantial vacancies in psychiatry specialties. Fill rates for core psychiatry training in Scotland have improved dramatically, filling at 100% since 2020. However, higher training fill rates have remained lower, with 68% filled in 2022. In response, the Scottish Government established a working group to examine the issues, both common and unique, to the whole psychiatry pipeline from training through to the consultant workforce, over two phases. The group aims to:

- Set out the current landscape.
- Consider the factors which influence recruitment and retention.
- Collate and analyse quantitative and qualitative evidence.
- Develop a set of recommendations.

**Methods:**

A representative group was convened, including SG Health Workforce and Mental Health Directorates, NHS Education for Scotland (NES), the Royal College of Psychiatrists Scotland, Health Board representatives (Associate Medical Directors, Clinical Directors, Directors of Medical Education) and trainee doctors. Representatives offer first-hand experience of training and working in psychiatry, knowledge and expertise in training programme management, workforce modelling data analysis, experience of a range of approaches to improve health workforce recruitment (including the use of financial incentives). The group has met 4 times since May 2023, with SG Health workforce directorate providing secretariat support.

**Results:**

Through the formation of this group, several areas affecting recruitment and retention were, and continue to be, addressed: enhanced exposure to psychiatry via FY1 simulation training, and increased number of FY2 psychiatry placements; the design and recruitment of clinical development fellow doctors; flexibility of training posts and the expansion of run through training programmes; using data to better support workforce modelling; trainee support, including tailored IMG support; the use of attraction campaigns and incentives in other devolved nations/specialties; alternative ways to provide clinical supervision; examining diversification of the MH workforce; international and domestic recruitment options.

**Conclusion:**

Several actions have been identified and progressed as the work of the group develops. Work is ongoing, and its impact will take time to emerge. This cross-functional group encouraged connectivity, conversation and network-building, striving to amplify differences and reduce power differentials, challenging traditional views. However, as with groups of this nature, there could be internal conflict where members fight strongly for ‘their corner’. Such a broad membership affects the development of a cohesive identity. Membership is largely voluntary, and so competing demands from the members’ existing responsibilities adds time pressure and stress, impacting commitment and productivity.

